# Smart city healthcare delivery innovations: a systematic review of essential technologies and indicators for developing nations

**DOI:** 10.1186/s12913-023-10200-8

**Published:** 2023-10-30

**Authors:** Zahra Mohammadzadeh, Hamid Reza Saeidnia, Aynaz Lotfata, Mohammad Hassanzadeh, Nasrin Ghiasi

**Affiliations:** 1 Health Information Management Research Center, Kashan University of Medical Sciences, Kashan, Iran; 2 Department of Health Information Management and Technology, School of Allied Medical Sciences, Kashan University of Medical Sciences, Kashan, Iran; 3 Department of Knowledge and Information Science, Tarbiat Modares University, Tehran, Iran; 4 School Of Veterinary Medicine, Department of Veterinary Pathology, University of California, Davis, USA; 5 Department of Public Health, School of Health, Ilam University of Medical Sciences, Ilam, Iran

**Keywords:** Smart City, Healthcare delivery, Smart health, Developing nations

## Abstract

**Background:**

In recent times, the concept of smart cities has gained remarkable traction globally, driven by the increasing interest in employing technology to address various urban challenges, particularly in the healthcare domain. Smart cities are proving to be transformative, utilizing an extensive array of technological tools and processes to improve healthcare accessibility, optimize patient outcomes, reduce costs, and enhance overall efficiency.

**Methods:**

This article delves into the profound impact of smart cities on the healthcare landscape and discusses its potential implications for the future of healthcare delivery. Moreover, the study explores the necessary infrastructure required for developing countries to establish smart cities capable of providing intelligent health and care services. To ensure a comprehensive analysis, we employed a well-structured search strategy across esteemed databases, including PubMed, OVID, EMBASE, Web of Science, and Scopus. The search scope encompassed articles published up to November 2022, resulting in a meticulous review of 22 relevant articles.

**Results:**

Our findings provide compelling evidence of the pivotal role that smart city technology plays in elevating healthcare delivery, forging a path towards improved accessibility, efficiency, and quality of care for communities worldwide. By harnessing the power of data analytics, Internet of Things (IoT) sensors, and mobile applications, smart cities are driving real-time health monitoring, early disease detection, and personalized treatment approaches.

**Conclusion:**

Smart cities possess the transformative potential to reshape healthcare practices, providing developing nations with invaluable opportunities to establish intelligent and adaptable healthcare systems customized to their distinct requirements and limitations. Moreover, the implementation of smart healthcare systems in developing nations can lead to enhanced healthcare accessibility and affordability, as the integration of technology can optimize resource allocation and improve the overall efficiency of healthcare services. It also may help alleviate the burden on overburdened healthcare facilities by streamlining patient care processes and reducing wait times, ensuring that medical attention reaches those in need more swiftly.

**Supplementary Information:**

The online version contains supplementary material available at 10.1186/s12913-023-10200-8.

## Introduction

A smart city represents a dynamic urban landscape that harnesses the power of technology and data-driven approaches to elevate the well-being and prosperity of its inhabitants [1, 2]. By seamlessly integrating cutting-edge technologies like sensors, the Internet of Things (IoT), automation, and artificial intelligence with various infrastructure systems, smart cities create a sustainable and highly efficient urban environment [3]. At its core, the main objective of a smart city is twofold: to optimize resource utilization and to enhance the quality of services and safety for its residents [4]. This vision is centered around a sophisticated information and communication technology system, acting as the backbone for seamless data collection, analysis, and distribution [5]. Through this interconnected infrastructure, smart cities can achieve intelligent decision-making and efficient resource management, creating a sustainable and improved urban living experience for their residents [2, 6, 7]. In addition to their technological advancements, smart cities actively involve their inhabitants in identifying challenges and collaboratively proposing solutions [8]. Through the strategic use of technology, they empower residents to play a meaningful role in the city’s development, fostering a stronger sense of community ownership and cohesion [9]. At the heart of smart cities is a firm commitment to sustainability, efficiency, and cooperation [10]. By prioritizing these elements, smart cities strive to enhance the quality of life for all residents while ensuring long-term economic growth in urban areas. Through continuous innovation and data-driven solutions, smart cities present a promising vision for the future, where urban life is redefined, and the potential for positive societal impact is maximized.

Numerous countries have achieved success in establishing smart cities that harness innovative technologies and data-driven approaches to enhance the well-being of their residents. Singapore, the Netherlands, and South Korea stand out as prominent examples in this domain [11]. Singapore, in particular, stands out as one of the most advanced smart cities globally, employing an array of technologies like sensors, analytics, and automation to enhance public transport efficiency, smart waste management, and environmental sustainability [12]. Amsterdam, the capital of the Netherlands, has made significant strides in environmental sustainability through projects involving electric vehicles and smart grid systems [13]. Additionally, the city has implemented sophisticated systems to monitor air pollution and waste levels, contributing to its reputation as a forward-thinking smart city. Seoul, the capital of South Korea, is another trailblazer in the realm of smart cities in Asia. Leveraging advanced technologies like the Internet of Things and big data, Seoul has made considerable progress in improving energy management, telemedicine, and e-government services [14]. These examples highlight how innovative solutions in smart cities have resulted in more sustainable, efficient, and livable urban environments worldwide. However, it is important to note that developing countries are also striving to create smart cities within their boundaries. Nevertheless, they face unique challenges in this endeavor [15]. Limited resources, infrastructural deficiencies, low technology adoption, and other factors present considerable obstacles for creating smart cities in these regions [16].

Despite the challenges, developing countries’ efforts to build smart cities are commendable and indicative of their determination to improve the lives of their residents through technological advancements. As technology becomes more accessible and innovative solutions are tailored to suit local needs, the potential for smart cities to flourish in developing nations grows, offering the promise of sustainable, inclusive, and prosperous urban environments for the future.

One of the key areas for smart city innovation is healthcare [17], where cities use a variety of technological tools and processes to improve access to care, improve patient outcomes, reduce costs, and increase efficiency. As urban populations continue to grow and healthcare demands increase, the smart city approach to healthcare is likely to become increasingly important in the coming years, offering new opportunities for both patients and healthcare providers [18].

The current body of research on the holistic impact of smart cities on healthcare delivery, especially in developing nations, remains insufficient and warrants further investigation. Through a comprehensive literature review, we aim to address the following research questions: (1) How can smart cities enhance healthcare delivery? (2) What are the technological tools employed by smart cities in advancing healthcare services? (3) What are the key indicators of a smart city’s healthcare capabilities, particularly in developing countries? To pursue these inquiries, we conducted a meticulous search using a well-structured strategy across reputable databases, namely PubMed, OVID, EMBASE, Web of Science, and Scopus. The search encompassed articles published up to November 2022 to ensure a comprehensive review of the relevant literature on the subject matter.

## Materials and methods

Our study involves a comprehensive literature review focused on examining the components and indicators related to Smart City applications in healthcare delivery. While preparing this manuscript, we have partially followed the PRISMA-ScR checklist, and the preferred reporting of systematic reviews as outlined by [19]. We have followed specific elements of the PRISMA-ScR checklist, such as:

1. ***Structured Summary***: Including background, objectives, eligibility criteria, evidence sources, diagramming methods, and conclusions related to the review questions and objectives. This helps provide a concise overview of the review.
2. ***Stating Questions and Objectives***: Clearly stating the questions and objectives that were raised in the review, with reference to their key elements. This helps readers understand the focus of the review.
3. ***Summarizing Main Results***: Providing a summary of the main results obtained from the review. This helps readers quickly grasp the key findings.
4. ***Interpretation of Results***: Providing a general interpretation of the results according to the questions and objectives of the review. This helps provide context and meaning to the findings.
5. ***Potential Implications***: Discussing potential implications of the review findings. This helps identify the practical implications and applications of the research.

It is worth noting that this manuscript has not been previously registered in PROSPERO or any equivalent database. We would like to clarify that the manuscript for this literature review has not been registered in PROSPERO or any equivalent database. While registration in PROSPERO is more commonly associated with systematic reviews, we made a conscious decision not to register this particular review. This decision was based on the scope of our review, which does not strictly adhere to the eligibility criteria of PROSPERO, and the feasibility within the constraints of our project. We assure readers that the rigorous methodology employed in our literature search and selection process, as well as the transparent reporting of our findings, are designed to address any concerns related to credibility.

### Inclusion and exclusion criteria

The study selection process involved specific inclusion criteria to identify relevant studies from the database. The following criteria were applied:

1) The article had to be either an original article or a short article published in English within a peer-reviewed journal. As a result, reports, letters to the editor, review articles, and meta-analyses were excluded from consideration. The rationale for excluding reports (often lack sufficient detail and rigor in methodology compared to peer-reviewed articles), letters to the editor (typically present opinions, comments, or responses to previously published articles, rather than original research), review articles (our aim was to focus on original research studies), and meta-analyses (often have their own inclusion and exclusion criteria, which may differ from ours).

2) The article had to focus on healthcare or smart health. Thus, articles solely addressing smart cities without any relevance to healthcare were excluded from the review.

By applying these criteria, we ensured that the selected studies were directly related to healthcare in the context of smart cities, providing a targeted and focused analysis for our research.

### Databases and search method

PubMed, OVID, EMBASE, Web of Science, and Scopus databases were searched for studies. In our search, we applied a date restriction from January 1, 2010, to November 2022. This timeframe was chosen to capture the most recent literature relevant to our research objectives. However, we did not impose any restrictions based on the start date of our search. This study used broad search terms and conducted a nested search [20]. To develop a comprehensive search strategy, we used a combination of keywords and controlled vocabulary terms (MeSH terms in PubMed) related to our research topic. The search terms included variations and synonyms to ensure maximum coverage. Here is an example of the search string used in PubMed: (“healthcare” OR “smart health”) AND (“technology” OR “digital health” OR “eHealth”) AND (“impact” OR “effectiveness” OR “outcomes” OR “evaluation”) AND (“Smart City” OR “Smart” OR “Smart-city” OR “Smart-cities”). By including relevant keywords and combining them using Boolean operators, we aimed to capture articles that focused on the impact, effectiveness, and evaluation of healthcare or smart health technologies. The specific search terms and string varied slightly for each database but followed a similar structure.

### Study selection

We conducted a two-step study selection process to identify articles that met our inclusion criteria. In the first step, two independent reviewers (HR.S. and Z.M.) screened the titles and abstracts of the identified articles to assess their relevance to our research question and inclusion criteria. Any disagreements between the reviewers were resolved through discussion and consensus. In cases where disagreements between the two reviewers persisted, a third reviewer (M.H.) was consulted as an arbitrator. The third reviewer carefully reviewed the articles in question and provided their input to reach a consensus. This process ensured that the final selection of articles was based on collective agreement.

### Study quality appraisal

The quality of included reviews was assessed by two researchers (HR.S. and Z.M.) using the CASP Systematic Review Checklist [21] (Appendix 1). We resolved any disagreements through discussion and agreed on the quality of the study.

## Results

**Fig. 1:**
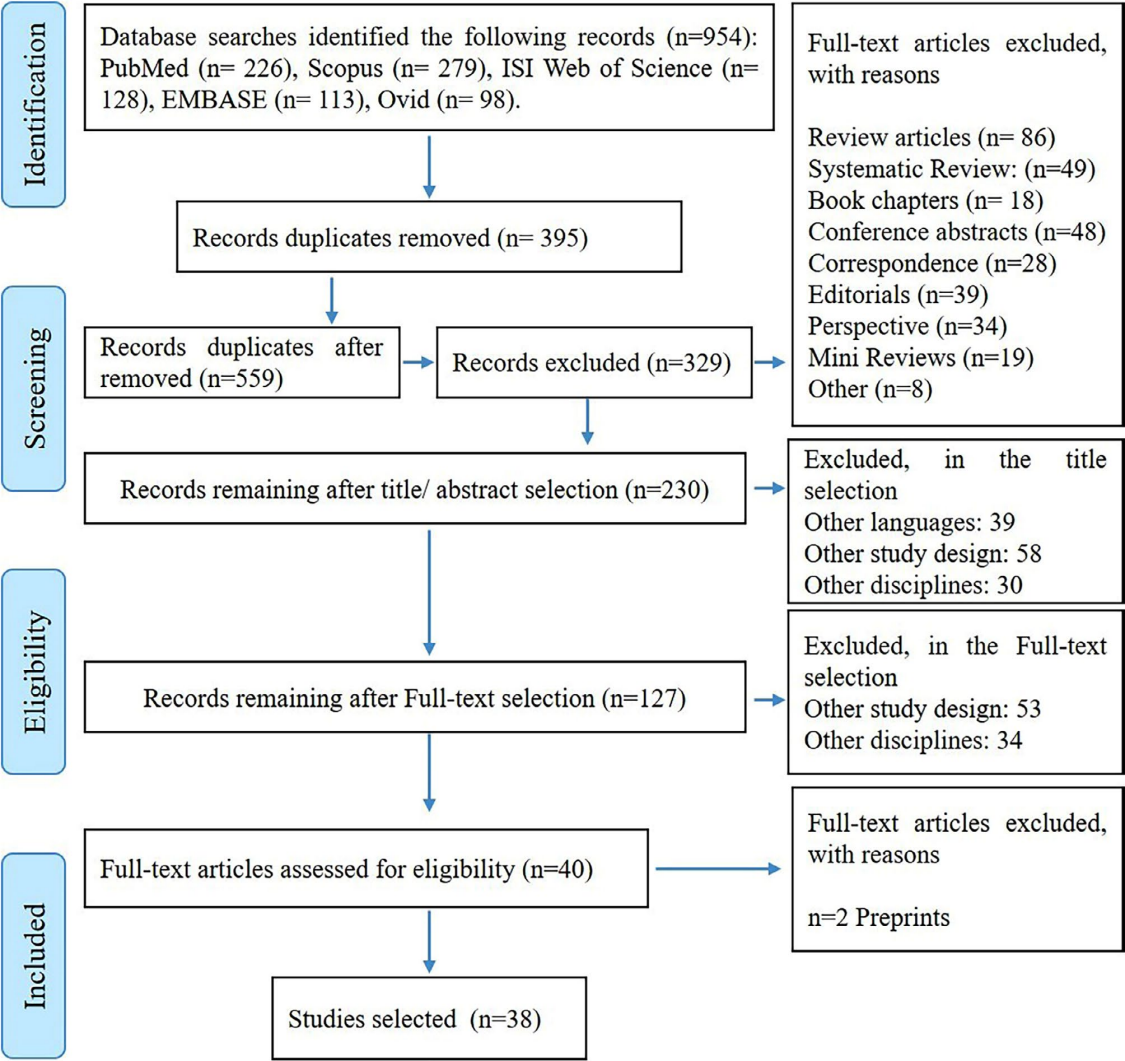
Flowchart of the review of the articles (PRISMA guidelines [19])

After conducting an initial search on databases including PubMed, OVID, EMBASE, Web of Science, and Scopus, a total of 954 articles were found. This marked the identification phase. After removing duplicates (381 articles) and articles without abstracts (14 articles) during the title and abstract screening, 559 articles remained for further evaluation. Based on specific criteria and a careful assessment of titles and abstracts, 329 articles were excluded. The abstracts of the remaining 230 articles were then thoroughly analyzed, resulting in the identification of 40 articles related to healthcare services in smart urban settings. However, 2 of these articles were preprints and not yet published, making them ineligible for the final analysis. Consequently, the final dataset consisted of 38 articles, as depicted in Fig. 1.

Based on the literature review, three research questions were addressed in this study:

### Research question 1) the incorporation of smart city approaches enhances healthcare delivery

**Fig. 2:**
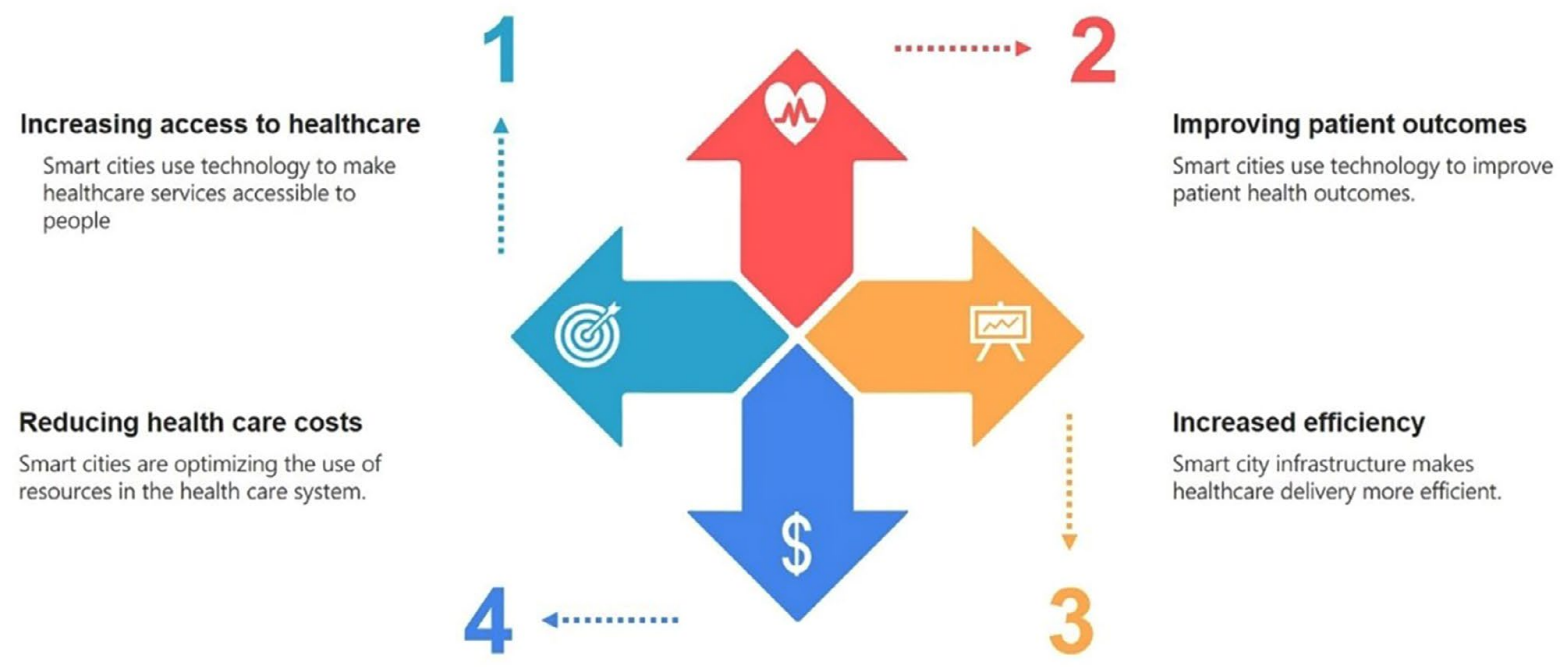
Illustrating research question 1 (How can smart cities enhance healthcare delivery?)

Based on the comprehensive review of the articles, it is evident that smart cities have the potential to revolutionize the healthcare sector through several key benefits. These include enhanced access to healthcare services, improved patient outcomes, reduced healthcare costs, and increased overall efficiency in healthcare delivery **(**Fig. 2**)**.

The question of how smart cities can enhance healthcare delivery has garnered significant attention, as evidenced by its emphasis in 12 separate studies. These studies have explored the potential benefits and strategies for leveraging smart city technologies to improve the quality, accessibility, and efficiency of healthcare services within urban environments (Table 1).

**Table 1:** Studies demonstrating how smart cities can enhance healthcare delivery

NO.	Author/s and Year	Main Point	Explanation of the point
1	Alahi et al., 2023; Lu & Deng, 2023; Talabani, 2022	Increasing access to healthcare	Smart cities use technology to make healthcare services accessible to people.
2	Alyami et al.; Badr, 2023a, 2023b	Improving patient outcomes	Smart cities use technology to improve patient health outcomes.
3	Cilke et al., 2004; González, 2022; Rentas González, 2022	Reducing health care costs	Smart cities are optimizing the use of resources in the health care system.
4	Braun et al., 2018; Johnson et al., 2020; Shamsuddin & Srinivasan, 2021	Increased efficiency	Smart city infrastructure makes healthcare delivery more efficient.

Smart cities have effectively addressed the challenge of healthcare accessibility through technological advancements, as evidenced by studies conducted by [22–24]. One notable approach is the implementation of telehealth services, allowing patients to receive medical consultations from remote locations. Moreover, smart city infrastructure has been utilized to enhance transportation options, ensuring easier and more convenient access to healthcare facilities for individuals.

Smart cities have demonstrated the ability to enhance patient health outcomes through the strategic application of technology, as indicated by studies conducted by [25–27]. A notable example is the utilization of wearable technology by healthcare providers to monitor patients’ health continuously and detect potential health issues at an early stage. This proactive approach to early disease prevention significantly contributes to improved patient outcomes and overall health.

Smart cities have effectively optimized resource utilization within the healthcare system, leading to significant reductions in healthcare costs, as evidenced by studies conducted by [28–30]. One notable approach is the integration of IoT devices, which enable real-time collection and analysis of healthcare data. This data-driven approach empowers healthcare providers to make more informed decisions, ultimately resulting in cost savings while maintaining the quality of care provided.

The implementation of smart city infrastructure has played a pivotal role in remarkably enhancing the efficiency of healthcare delivery, as demonstrated by studies conducted by [31–33]. An illustrative example of this efficiency is the accessibility of electronic health records (EHR), enabling healthcare providers to access real-time patient information swiftly and seamlessly. This streamlined access to critical data empowers healthcare professionals to make informed decisions promptly, leading to more efficient and effective healthcare services for patients.

### Research question 2) Technological tools employed by smart cities in advancing healthcare services

**Fig. 3:**
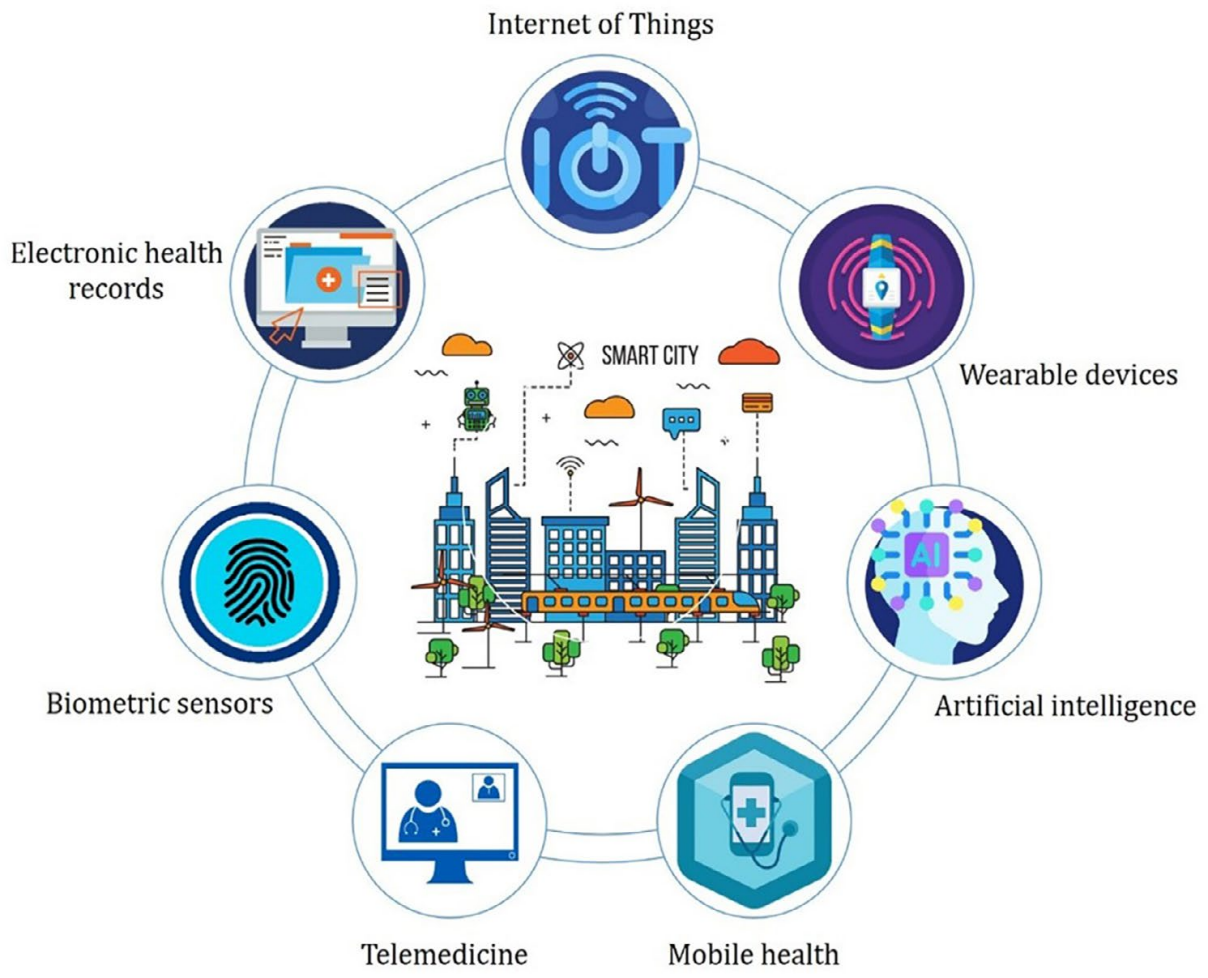
Illustrating fundamental technologies for a smart city in providing health services

Through a comprehensive review of the collected articles, it becomes evident that the fundamental technologies underpinning a smart city’s healthcare services are IoT devices, Wearable devices, Artificial Intelligence (AI), Telemedicine, EHRs, Mobile Health (mHealth), and Biometric sensors **(**Fig. 3**)**. These technologies are at the forefront of shaping the future of healthcare in smart cities, offering innovative solutions to enhance patient care, monitoring, and overall health management.

The question of how smart cities utilize technological tools to advance healthcare services has attracted significant attention, as indicated by its prominence in 22 distinct studies (Table 2*). These studies have explored the various technological tools employed by smart cities to enhance healthcare delivery. Through their research, they have identified and examined a range of innovative technologies that smart cities utilize in the healthcare sector (*Table 2*).*

**Table 2:** Studies that demonstrate how smart cities use technological tools to advance healthcare services

NO.	Author/s and Year	Main Point	Explanation of the point
1	Alavi et al., 2018; Almalki et al., 2021; Bauer et al., 2021; Syed et al., 2021	Internet of Things (IoT) devices	These devices can be used to remotely monitor patients and provide real-time information about their health status
2	Balsamo et al., 2017; Weddell & Magno, 2018	Wearable devices	These devices can monitor vital signs such as heart rate, blood pressure and oxygen levels and send this information to healthcare providers for analysis
3	Allam & Dhunny, 2019; Cugurullo, 2020; Luckey et al., 2021; Nikitas et al., 2020	Artificial Intelligence (AI)	Artificial intelligence can be used to analyze healthcare data and provide insights that can help in timely diagnosis and treatment of diseases
4	Ahmed et al., 2023; Hassankhani et al., 2021; Sahatiya & Singh, 2023	Telemedicine	Telemedicine allows patients to consult with healthcare providers remotely, avoiding the need for travel and reducing the risk of infection
5	Al Omar et al., 2021; Parah et al., 2020	Electronic Health Records (EHRs)	EHRs allow healthcare providers to access patient data from anywhere, enabling more efficient and coordinated care
6	Al-Azzam & Alazzam, 2019; Butt et al., 2022; Istepanian & AlAnzi, 2020; Lotfi et al., 2020	Mobile Health (mHealth)	mHealth apps can help patients manage chronic diseases and track their health progress, allowing healthcare providers to monitor their patients more closely
7	Golec et al., 2020; Obaidat et al., 2019; Ross et al., 2020	Biometric sensors	Biometric sensors can be used to monitor patients’ movements and alert healthcare providers if there is any unusual activity, such as falls or seizures

IoT devices have emerged as valuable tools for remote patient monitoring, allowing healthcare providers to access real-time information about their patients’ health status. By enabling seamless connectivity and data exchange, IoT devices play a crucial role in enhancing healthcare delivery and improving patient outcomes [5, 34–37].

Wearable devices have proven to be invaluable in monitoring essential vital signs such as heart rate, blood pressure, and oxygen levels. These devices efficiently transmit this critical information to healthcare providers for analysis, enabling timely and informed decision-making to ensure patients receive the best possible care and attention [38–40].

AI has demonstrated remarkable capabilities in analyzing healthcare data, offering invaluable insights that contribute to timely disease diagnosis and treatment. By harnessing the power of AI, healthcare professionals can make more informed decisions, leading to more effective and efficient healthcare interventions for patients [41–45].

Telemedicine has emerged as a transformative approach, enabling patients to consult with healthcare providers remotely. By eliminating the need for travel, telemedicine not only enhances convenience but also effectively reduces the risk of infection, contributing to a safer and more accessible healthcare experience for patients [46–49].

EHRs have revolutionized healthcare by providing healthcare providers with the ability to access patient data from anywhere. This seamless accessibility facilitates more efficient and coordinated care, ensuring that healthcare professionals have the most up-to-date information to make well-informed decisions and deliver optimal patient outcomes [50, 51].

mHealth applications have proven to be invaluable in empowering patients to manage chronic diseases and monitor their health progress proactively. These apps enable patients to track vital health metrics and share this data with healthcare providers, facilitating closer monitoring and personalized care. Through mHealth solutions, healthcare professionals can stay closely connected with their patients, leading to improved health outcomes and more effective disease management [6, 52–55].

Biometric sensors offer a valuable means of monitoring patients’ movements, capable of alerting healthcare providers promptly in the event of any unusual activity, such as falls or seizures. These sensors play a crucial role in enhancing patient safety and enabling healthcare professionals to respond swiftly to potential health emergencies, ensuring timely and appropriate interventions for improved patient care [56–58].

### Research question 3) indicators of a smart city for Healthcare in a developing country

**Fig. 4:**
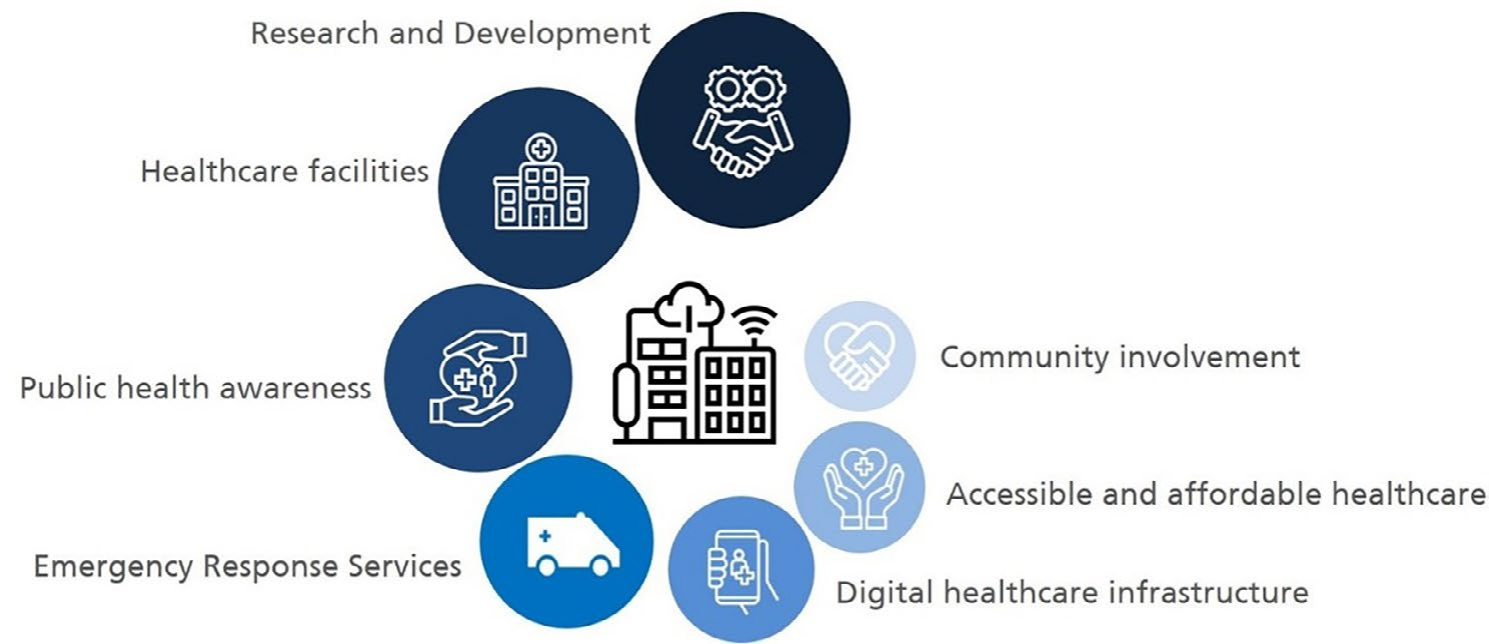
Indicators of a smart city for healthcare in a developing country

In a developing country, the indicators of a smart city for healthcare may indeed vary based on the country’s specific needs and circumstances **(**Fig. 4**)**. However, several key indicators could be considered when evaluating the progress of a smart city in enhancing healthcare services. Some of these indicators include:

The question of identifying the key indicators of a smart city’s healthcare capabilities, specifically in developing countries, has received considerable attention, evident from its prevalence in 16 separate studies (Table 3). These studies have focused on understanding and analyzing the specific indicators that determine the effectiveness and efficiency of healthcare services in smart cities within developing countries.

**Table 3:** Studies that demonstrate identifying the key indicators of a smart city’s healthcare capabilities in developing countries

NO.	Author/s and Year	Main Point	Explanation of the point
1	Kaluarachchi, 2022; Neffati et al., 2021	Accessible and affordable healthcare	A smart city for healthcare in a developing country should focus on providing affordable and accessible healthcare to all citizens
2	Ross et al., 2020; Shamsuddin & Srinivasan, 2021; Syed et al., 2021	Digital healthcare infrastructure	A smart city for healthcare must have a robust digital infrastructure to enable the smooth flow of data between healthcare providers and patients
3	Angelidou, 2014; Anthopoulos, 2015; Braun et al., 2018	Healthcare facilities	In a developing country, a smart city for healthcare should have hospitals and clinics that are equipped with the latest medical technology, diagnostic equipment, and trained healthcare professionals
4	Al-Azzam & Alazzam, 2019; Alahi et al., 2023; Allam & Dhunny, 2019	Public health awareness	A smart city for health care should have a system that increases public health awareness and encourages citizens to adopt healthy lifestyle habits and reduce the risk of diseases
5	(Al-Azzam & Alazzam, 2019; Syed et al., 2021	Emergency Response Services	A smart city for healthcare should have an efficient emergency response system including ambulance services and emergency medical technicians to provide timely assistance to patients
6	Nikitas et al., 2020; Obaidat et al., 2019; Shamsuddin & Srinivasan, 2021	Research and Development	In developing countries, a smart city for healthcare should focus on research and development to find innovative solutions to address public health challenges
7	(González, 2022; Granier & Kudo, 2016; Hassankhani et al., 2021	Community involvement	A smart city for healthcare should encourage community involvement and participation, empowering local people to take ownership of their health and well-being and fostering a sense of social responsibility for health

A smart city for healthcare in a developing country must prioritize the provision of accessible and affordable healthcare services to all residents [1, 59]. This encompasses not only basic healthcare services and treatments but also preventive measures such as immunizations. By emphasizing affordability and accessibility, the smart city can work towards improving the overall health and well-being of its population, ensuring that essential healthcare is within reach for everyone, regardless of their socio-economic status.

A smart city for healthcare necessitates a robust digital infrastructure to facilitate seamless data flow between healthcare providers and patients [31, 35, 56]. Essential components of this digital healthcare infrastructure include electronic health records, telemedicine facilities, and mobile health applications. By establishing these digital platforms, the smart city can enhance communication, improve access to medical information, and enable remote healthcare services, ultimately promoting more efficient and effective healthcare delivery for its residents.

In a developing country, a smart city for healthcare should prioritize well-equipped hospitals and clinics with the latest medical technology, advanced diagnostic equipment, and a skilled workforce of healthcare professionals [2, 3, 32]. By ensuring access to cutting-edge medical resources and expert care, the smart city can significantly improve the quality and efficacy of healthcare services, catering to the diverse healthcare needs of its population and enhancing overall health outcomes.

A smart city for healthcare must incorporate a system that fosters public health awareness and encourages residents to adopt healthy lifestyle habits, reducing the risk of diseases [6, 24, 41]. By actively promoting health awareness campaigns and leveraging technology to disseminate vital health information, the smart city can empower its residents to make informed decisions about their well-being, leading to a healthier and more resilient population.

A smart city for healthcare must prioritize the establishment of an efficient emergency response system, comprising ambulance services and well-trained emergency medical technicians, to ensure timely assistance to patients in need [6, 35]. By swiftly attending to medical emergencies, the smart city can enhance the chances of positive health outcomes and reinforce the safety and well-being of its residents.

In developing countries, a smart city for healthcare must place a strong emphasis on research and development to discover innovative solutions that effectively address public health challenges [31, 45, 57]. By actively investing in research endeavors and fostering collaboration between academia, healthcare institutions, and technology experts, the smart city can pave the way for transformative advancements in healthcare delivery, ultimately benefiting its residents and contributing to improved health outcomes for the entire population.

A smart city for healthcare should actively promote community involvement and participation, empowering local residents to take charge of their health and well-being while fostering a sense of social responsibility for health [9, 28, 48]. By encouraging active engagement from the community, the smart city can establish a collaborative environment where residents play an integral role in shaping healthcare initiatives, leading to a more inclusive and responsive healthcare system that addresses the unique needs of the population and ultimately improves overall health outcomes.

## Discussion

Regarding the first research question (i.e., How can smart cities improve healthcare), four key aspects of application emerged as significant contributors: (1) Increasing access to healthcare (4 articles); (2) Improving patient outcomes (5 articles); (3) Smart cities using technology to enhance patient health outcomes (3 articles); (4) Increased efficiency (4 articles) Overall, smart cities are at the forefront of healthcare innovation, driving advancements in access, patient outcomes, cost reduction, and efficiency. The conclusive findings highlight enhanced patient experiences and the seamless integration of technology into healthcare delivery, indicating a promising trajectory for the future of healthcare in smart cities.

Regarding the second research question (i.e., Technologies used by a smart city in providing healthcare), the review identified seven crucial and practical aspects, supported by the following number of articles: (1) Internet of Things devices (4 articles); (2) Wearable devices (3 articles); (3) Artificial Intelligence (5 articles); (4) Telemedicine (4 articles); (5) Electronic Health Records (4 articles); (6) Mobile Health (10 articles); (7) Biometric sensors (3 articles).

These technologies showcase the significant role of innovation in smart cities, highlighting their potential to revolutionize healthcare delivery and improve patient outcomes through cutting-edge applications and solutions.

Regarding the third research question (i.e., What are the indicators of a smart city for healthcare in a developing country), the review identified seven essential and functional aspects, supported by the following number of articles:1. Accessible and affordable healthcare (3 articles); 2. Digital healthcare infrastructure (4 articles); 3. Healthcare facilities (5 articles); 4. Public health awareness (5 articles); 5. Emergency Response Services (3 articles); 6. Research and Development (4 articles); 7. Community involvement (2 articles).

These indicators provide valuable insights into the key areas that a smart city in a developing country should prioritize to enhance healthcare accessibility, quality, and overall public health outcomes. By focusing on these aspects, a smart city can effectively address the unique challenges faced by developing nations, leading to improved healthcare services and better health outcomes for its residents.

Smart city technology plays a crucial role in enhancing healthcare by improving access, efficiency, and quality [60]. Among the effective technologies, data analysis and IoT sensors enable the monitoring of community health, with predictive analysis aiding in early diagnosis and interventions [61]. An essential indicator for a smart city in healthcare delivery involves the seamless collection and integration of data from diverse sources, including electronic health records, wearable devices, social media, and environmental sensors [24, 50]. To ensure data security and protect individuals’ personal information, data governance and privacy regulations must be implemented, while addressing ethical and regulatory issues such as bias, transparency, and accountability [53]. Additionally, a critical infrastructure in developing a smart city for healthcare is the adoption of telemedicine and virtual care [47, 49]. The COVID-19 pandemic accelerated this transition, showcasing the high functionality of these digital health technologies and their potential to improve access to care [49]. By embracing these advancements, smart cities can advance their healthcare capabilities, providing more efficient, accessible, and patient-centered services for their communities.

Engaging healthcare providers, patients, policymakers, and technology developers in smart cities is crucial for aligning healthcare technology with community needs and values [33, 45, 59]. Adopting a multidisciplinary strategy encompassing technical feasibility, social impact, economic viability, and environmental sustainability is essential [29, 42]. By fostering collaboration among diverse stakeholders, smart cities can create healthcare solutions that are not only innovative and efficient but also responsive to the unique requirements and aspirations of their communities.

A smart city’s success relies on a robust digital infrastructure, underpinned by a strong and stable internet connection. For developing countries, investing in broadband connectivity is imperative to enable residents’ access to essential digital services like e-government, e-health, and e-education [62]. Moreover, these countries must prioritize resident participation in health-oriented policies to ensure the effective implementation of smart city initiatives [63]. Smart cities offer residents a unique opportunity to collaborate with local governments, actively participating in decision-making and urban planning endeavors [59]. Developing countries should emphasize resident engagement to ensure their smart city programs cater to the needs of their residents, accurately reflecting their aspirations and empowering them to take an active role in project planning and implementation [15]. By doing so, these countries can develop a unique and tailored approach that best addresses their existing challenges and priorities, while adhering to global smart city standards. In this way, they can effectively leverage smart city technologies to enhance the overall quality of life for their residents and foster sustainable urban development.

Smart city technology has brought about a transformative impact on healthcare delivery, significantly enhancing access, efficiency, and the overall quality of care. Policymakers play a crucial role in the successful implementation of healthcare-related smart city initiatives, ensuring that data and technology are used ethically to safeguard patient privacy and data security. The integration of machine learning algorithms has enabled the analysis of vast healthcare data, enabling the identification of patterns and early signs of diseases for proactive interventions. Furthermore, wearable fitness trackers and sensors play a crucial role in detecting warning signs of chronic diseases at an early stage, promoting timely medical interventions. Smart city technology has also proved invaluable in public health management by continuously monitoring air and water quality. Policymakers have developed comprehensive policies for regular environmental data reporting, fostering healthier urban environments for the benefit of residents. With access to real-time data on disease outbreaks, healthcare providers can respond proactively, swiftly curbing the spread of illnesses. This proactive approach significantly contributes to improving public health outcomes and reducing the burden on healthcare facilities. Moreover, smart city technology optimizes healthcare services by efficiently managing resources and reducing patient waiting times. This leads to an overall improvement in the healthcare experience for individuals and facilitates better healthcare outcomes. The involvement of policymakers in the development and implementation of smart city technology is instrumental in reshaping healthcare delivery. Through their support, personalized, efficient, and accessible care can be delivered to a broader population while effectively managing costs. In this fast-evolving landscape, continuous innovation and collaboration between technology experts, healthcare professionals, and policymakers will be crucial in shaping the future of healthcare within smart cities. By harnessing the full potential of smart city technology, we can revolutionize healthcare and ensure a healthier and more connected world.

### Implications of healthcare delivery innovation in developing countries

Implementing healthcare delivery innovation in developing countries requires a strategic and holistic approach that takes into account the unique challenges and opportunities these countries face. Here are some key steps to consider:

#### Identify local needs and challenges

Conduct a thorough assessment of the healthcare landscape in the specific developing country to identify the most pressing healthcare needs and challenges. This will help prioritize areas that require innovation and improvement.

#### Foster Public-private partnerships

Collaboration between the government, private sector, non-governmental organizations (NGOs), and other stakeholders is essential for successful healthcare innovation. Public-private partnerships can pool resources, expertise, and technology to develop and implement innovative solutions.

#### Leverage mHealth and telemedicine

Given the widespread access to mobile phones in many developing countries, mHealth and telemedicine solutions can significantly improve healthcare access. Implementing mobile health apps and telemedicine platforms can enhance remote consultations, health monitoring, and health education.

#### Enhance Healthcare infrastructure

Investing in healthcare infrastructure, such as building and upgrading hospitals and clinics, ensures that the necessary facilities are available to support innovative healthcare delivery.

#### Train Healthcare professionals

Provide training and education for healthcare professionals on using innovative technologies and approaches. This will ensure that they can effectively implement and utilize new healthcare delivery methods.

#### Emphasize Preventive Healthcare

Healthcare innovation should focus on preventive measures to address public health issues effectively. Initiatives such as immunization campaigns, health screenings, and health awareness programs can have a significant impact on reducing disease burdens.

#### Integrate EHRs

Implementing EHR systems can streamline patient data management and improve healthcare coordination, leading to more efficient and patient-centered care.

Implement Remote Monitoring and IoT Devices: Remote monitoring devices and Internet of Things (IoT) technology can help in real-time health monitoring, especially for chronic disease management. These technologies can be particularly beneficial in rural and underserved areas.

#### Promote Health Information sharing

Encourage the sharing of health information and best practices among healthcare providers and institutions to improve decision-making and patient outcomes.

#### Address Regulatory and Policy barriers

Review and revise existing policies and regulations to facilitate the adoption of innovative healthcare technologies and practices. This includes ensuring data privacy and security regulations are in place.

#### Focus on affordability and sustainability

Ensure that healthcare innovations are affordable and sustainable in the local context. Consider cost-effective solutions that can be scaled up to reach a broader population.

By taking a comprehensive and context-specific approach, healthcare delivery innovation can significantly improve healthcare access, quality, and outcomes in developing countries, ultimately contributing to better health and well-being for their populations.

### Comparative analysis of developing nations with developed nations

Smart city initiatives in developed nations incorporate technologies like smart grids, intelligent transportation systems, and automated waste management, improving sustainability, efficiency, and quality of life. These nations also allocate significant resources to research and development (R&D), fostering innovation, technological advancements, and economic growth.

Developed nations prioritize environmental sustainability by investing in renewable energy, promoting green initiatives, and implementing strict environmental regulations. This focus on sustainability yields long-term economic and environmental benefits. Digital government services, such as online tax filing, e-voting, and digital identity systems, streamline administrative processes, enhance citizen engagement, and improve government efficiency.

Quality education systems in developed nations provide access to comprehensive and well-funded education. These systems leverage technology in classrooms, offer specialized programs, and emphasize continuous skill development. Developed nations also have efficient transportation systems, including high-speed railways, well-maintained roads, and advanced public transportation networks, enhancing connectivity, reducing congestion, and improving logistics.

Fintech innovations are prominent in developed nations, with digital payment systems, mobile banking, and blockchain technology. These advancements improve financial services, increase financial inclusion, and support economic growth. Additionally, developed nations leverage data analytics and artificial intelligence for informed policy decisions. They have robust data infrastructure, open data initiatives, and data-driven governance models.

Comparing these additional technologies and indicators with those mentioned earlier for developing nations highlights the disparities and areas for improvement. It also identifies potential opportunities for collaboration and knowledge sharing between developed and developing nations, enabling the transfer of expertise, resources, and best practices.

### Practical implications and recommendations

The findings of this review have several practical implications for policymakers and stakeholders in developing countries. Firstly, the identified studies consistently demonstrate the potential of healthcare or smart health technologies to improve healthcare delivery and outcomes. This highlights the importance of considering the integration of these technologies into existing healthcare systems.

Secondly, the review findings suggest that healthcare or smart health technologies can address specific challenges faced by developing countries, such as limited access to healthcare services, resource constraints, and inadequate healthcare infrastructure. By leveraging these technologies, policymakers can potentially overcome these barriers and improve healthcare access and delivery in resource-limited settings.

Furthermore, the review highlights the need for policymakers to prioritize investment in digital health infrastructure and capacity building. Developing countries should invest in robust information technology systems, secure data management protocols, and training programs to ensure the successful implementation and sustainable use of healthcare technologies.

### Recommendations

1. Policy Support: Policymakers should develop and implement policies that support the integration of healthcare or smart health technologies into existing healthcare systems. This includes creating regulatory frameworks, guidelines, and standards to ensure the safety, privacy, and interoperability of these technologies.
2. Infrastructure Development: Policymakers should prioritize the development of digital health infrastructure, including internet connectivity, telecommunication networks, and electronic health records systems. This will facilitate the seamless exchange of health information and enable the effective implementation of healthcare technologies.
3. Capacity Building: Policymakers should invest in training programs to enhance the digital health literacy of healthcare providers, administrators, and other stakeholders. This will ensure that they have the necessary skills to effectively utilize healthcare technologies and maximize their potential benefits.
4. Public-Private Partnerships: Policymakers should foster collaborations between the public and private sectors to leverage expertise, resources, and innovative solutions in the development and implementation of healthcare technologies. Public-private partnerships can help overcome financial constraints and accelerate the adoption of these technologies.
5. Monitoring and Evaluation: Policymakers should establish mechanisms for monitoring and evaluating the impact and effectiveness of healthcare technologies. This will enable evidence-based decision-making and continuous improvement in the implementation and scaling up of these technologies.

By considering these recommendations, policymakers and stakeholders in developing countries can harness the potential of healthcare or smart health technologies to address healthcare challenges and improve health outcomes for their populations.

### Limitations

It is important to acknowledge the limitations of our study, as they may impact the comprehensiveness of our findings. Firstly, the selected keywords and databases used in our study were constrained, potentially limiting the scope of the literature reviewed. Despite our efforts to include a comprehensive set of keywords and synonyms, it is possible that some relevant studies may have been missed due to variations in terminology or the use of different keywords that were not included in our search strategy. This could affect the comprehensiveness of the literature review and potentially result in the omission of relevant studies. Moreover, our access to certain databases was restricted, leading to potential gaps in the research we could access. Furthermore, we did not include the gray literature in our review, which represents an important gap in our study. Local field projects and initiatives, which could be valuable in understanding smart city healthcare developments, may not be published in scientific papers and thus were not considered in our analysis. Despite these limitations, our study offers valuable insights into the role of smart city technology in healthcare. However, it is essential to consider these limitations when interpreting the results, and future research could address these issues to provide a more comprehensive understanding of the subject matter.

## Conclusion

The primary objective of this research study was to identify crucial technologies and infrastructures relevant to a developing country and determine fundamental indicators for a smart city in healthcare delivery services. To achieve this goal, we conducted a comprehensive review of the existing literature, analyzing the insights and solutions presented by various researchers. Through a thorough examination of the retrieved articles, we interpreted and analyzed the technologies, infrastructures, and indicators impacting healthcare delivery. Our study offers valuable information to smart city practitioners and researchers alike, shedding light on the significance of smart city technologies and infrastructure, particularly in improving healthcare services in developing countries. By identifying essential indicators, our research contributes to the development and implementation of effective healthcare strategies within the context of smart cities.

### Electronic supplementary material

Below is the link to the electronic supplementary material.

Supplementary Material 1

## Data Availability

Please contact the corresponding author if you would like access to the datasets used and/or analyzed during this study.
